# Advances in culture methods for acute myeloid leukemia research

**DOI:** 10.18632/oncoscience.540

**Published:** 2021-08-04

**Authors:** Krishnapriya Syama, Eman M. Hassan, Shan Zou

**Affiliations:** ^1^Metrology Research Centre, National Research Council Canada, Ottawa, Ontario, Canada

**Keywords:** co-culture, 3D culture, CD47, calreticulin, cytokines

## Abstract

Conventional suspension cultures have been used in Acute Myeloid Leukemia (AML) research to study its biology as well as to screen any drug molecules, since its inception. Co-culture models of AML cells and other stromal cells as well as 3 dimensional (3D) culture models have gained much attention recently. These culture models try to recapitulate the tumour microenvironment and are found to be more suitable than suspension cultures. Though animal models are being used, they require more time, effort and facilities and hence, it is essential to develop cell culture models for high-throughput screening of drugs. Here, we discuss a new co-culture model developed by our research group involving acute myeloid leukemia (AML) cells and stimulated macrophages. Other studies on co-culture systems and relevance of 3D culture in leukemic research in understanding the pathology and treatment of leukemia are also reviewed.

## INTRODUCTION

Immune therapy has been a focus of research for past few decades [1, 2]. There are various strategies to modulate the immune response – functional monoclonal antibodies as immune checkpoint inhibitors, cell based therapies such as chimeric antigen receptor (CAR)-T cell therapy, inhibition of immunosuppressive mechanisms and vaccines to improve antigen presentation [3–7]. These approaches are being used in the treatment of hematological malignancies [8, 9]. Conventional 2D culture of leukemia cells involve only tumour cells in suspension and they are not in communication with other stromal cells or immune cells, unlike in a tumour microenvironment [10]. They also lack cell-extra cellular matrix (ECM) interactions [11]. Xenograft *in vivo* models are considered to be the gold standard in cancer research, however, it requires more time, effort and facilities and therefore more expensive [12, 13]. Hence, *in vitro* models need to be modified, which include the tumour – immune cell interactions.

In a tumour microenvironment, malignant cells are in contact with different types of cells like non-malignant stromal cells, macrophages and T lymphocytes [14–16]. They are continuously engaged in cytokine, chemokine and other growth factor signaling cascades, which helps in their proliferation, migration and invasion [17, 18]. Hence it is difficult to exactly recapitulate the *in vivo* tumour microenvironment *in vitro*. The difference in signaling mechanisms contributing for leukemic cell proliferation in both *in vitro* and *in vivo* models has been reviewed very recently [19]. These differences should be considered to develop more reliable *in vitro* models.

Co-culture models have been gaining much attention in cancer research due to their recapitulation of the tumour microenvironment. Co-culture models are broadly of 2 types: 1) Direct and 2) Indirect [20, 21]. In direct co-culture, two types of cells, for example; tumour cells and stromal cells, are physically in contact with each other and communicate through paracrine signaling. However, in an indirect co-culture, tumour cells and stromal cells are cultured in separate compartments or conditioned medium of one cell type is supplied to the other cell type. This allows the sharing of biomolecules through a permeable membrane, which segregates the two types of cells [20, 22]. From our perspective, direct co-culture seems to be better as it mimics the conditions in an *in vivo* tumour. In a tumour microenvironment, cancer cells are surrounded by stromal cells and cancer-stromal cell interactions are crucial for tumorigenesis and metastasis. Direct co-culture would be satisfying the above *in vivo* conditions, hence it may be better than indirect co-culture models. Co-culture models with tumour cells and stromal cells are considered to be better to evaluate therapeutic drugs like monoclonal antibodies, small molecule inhibitors and nano-drug carriers [23]. Some of the highlights and complications of these co-culture approaches are summarized in Table 1.

**Table 1:** 2D and 3D co-culture methods for the leukemia research

**Method**		**Analysis Type**	**Highlights**	**Complications**	**Examples**
**2D culture**		Cells are grown as suspension cells	Easy and cheap compared to 3D culture methods	Lack cell-cell junctions, sensitive to all drugs, gene and protein expression levels are different to that of *in vivo* tumours	AML [77, 78]
**3D culture**		Cells are grown as spherical-like structures on different matrices/scaffolds and have multiple layers	Close to the *in vivo* tumour microenvironment, and cells are resistant to drugs and hence physiologically relevant Cell-extra cellular matrix interactions can be studied	Expensive Time consuming Needs optimization	AML [61, 79, 80]
**Co-culture**	**Direct co-culture**	Two types of cells – Tumour and stromal; Both tumour and stromal cells are physically in contact with each other	Can study the effect of stromal cells on tumour cells and *vice-versa*	Unable to do downstream assays separately for each type of cell	AML and mesenchymal stromal cells [81] Mesenchymal stem and AML cells [61]
	**Indirect co-culture**	In separate compartments; Medium from one cell type is supplied to the other cell type	Sharing of biomolecules through a permeable membrane Better evaluate therapeutic drugs than a 2D culture	Permeable membrane inserts are expensive and these models do not exactly mimic the conditions in a tumour microenvironment	AML cells and macrophages [24]
	**3D co-culture**	Leukemia cells are grown as 3D structures and grown with stromal/immune cells	Mimic more closely the histologic conditions compared to 2D co-culture It can be an intermediate platform between 2D and xenografts	Expensive and need to optimize the matrices/scaffolds for both tumour and stromal cells	AML and bone marrow mononuclear cells [79] Human bone marrow mesenchymal stem and AML cells [61]

### Co-culture model with AML cells and macrophages

Our research group has recently demonstrated the use of a co-culture model with Acute Myeloid Leukemia (AML) cells and stimulated macrophages *in vitro* [24]. This study demonstrated that AML cells, when in co-culture with stimulated macrophages, were eliminated and their expression of the “don’t eat me signal”, CD47 [25–28] was down-regulated.

This study showed that CD47 inhibition was successful and selective in AML but not normal cells [24]. Expression levels of another protein, Calreticulin (CRT), “eat me signal” were up-regulated in the AML cells co-cultured with stimulated macrophages. Interestingly, significant down-regulation of CD47 and up-regulation of CRT expression was observed in AML cells, only when they are co-cultured with stimulated macrophages and not under any other culture conditions. Activated macrophages were shown to secrete high levels of cytokines such as IL-12p70, IL-6 and TNF-α. This co-culture model can be used to screen the efficacy of new drugs in AML treatment. It also demonstrates the possibility of using human macrophages for the treatment of AML [24]. There are only a few reports focusing on restoring immune surveillance in AML cells. Some of the studies were either focused on inhibition of CD47 or on up-regulation of CRT [29–31]. The co-culture model developed by our group, demonstrated that stimulated macrophages help both phenomenon to occur simultaneously [24].

Various strategies have been used to target CD47, as it serves as the “don’t eat me signal”, and inhibit phagocytosis by macrophages, thereby reducing the immune surveillance. Immunotherapy targeting CD47-SIRP-α are under clinical investigation, mainly monoclonal antibodies [32–34]. One of the monoclonal antibodies, 5F9, has been showing promising results in a phase 1b clinical trial and currently, Hu5F9/magrolimab combined with azacitidine (placebo as control) is in phase III trial [35]. Small molecule inhibitors and tyrosine kinase inhibitors were shown to target CD47-SIRP-α signaling in leukemia, lymphoma and melanoma cells [36, 37]. Other molecules such as Pep20, D4-2, RRx-001, metformin were reported to block this signaling axis in colon carcinoma, lymphoma, melanoma, non-small cell lung cancer and breast cancer [37]. The co-culture model developed with AML cells and macrophages is a good platform to test these monoclonal antibodies (mAbs) and small molecule inhibitors. By adding different concentrations of mAbs or small molecule inhibitors to the tumour cells in co-culture with macrophages, the expression of CD47 and CRT can be evaluated by flow cytometry. We can also evaluate if these drugs promotes the elimination of tumour cells by apoptosis or viability assays such as Alamar blue assays. The synergistic effect of stimulated macrophages and the drug molecules can be evaluated using this co-culture model. The successful co-culture studies could be extended to *in vivo* mouse models.

### Drug screening using two dimensional (2D) co-culture models of leukemia

Co-culture studies with leukemic cells and stromal cells or immune cells led to the identification of many drug targets [38]. The first study to report the co-culture of leukemia cells and bone marrow derived stromal cells demonstrated that purified chronic lymphocytic leukemia (CLL) cells grown on stromal cells had a prolonged survival [38, 39]. It was also demonstrated that CLL –marrow stromal cell co-culture offer a more reliable and relevant model to study the marrow stromal cell – CLL cell interactions, when compared to suspension cultures [40]. In another report, HS-5 stromal cells were pre-treated with anti-cancer drugs like Ara-C, doxorubicin, daunorubicin and then, co-cultured with K562 cells. In co-culture, K562 cells proliferated rapidly and hence, it can be inferred that leukemia patients when undergoing chemotherapy have deficient stromal cells resulting in a cytokine-deficient microenvironment. These aspects should be considered during chemotherapeutic failure [41].

Another preclinical study demonstrated the potential of CD4CAR-expressing T cells in eliminating malignant CD4+ cells. It was shown that CD4-specific chimeric antigen receptor (CAR)-engineered T cells (CD4CAR T cells), when in co-culture with CD4+ T cell leukemic cell lines (KARPAS 299 cells, primary leukemia cells from a patient), eliminated CD4+ T leukemic cells [42]. Another report showed that on co-culture with bone marrow stromal cells, MS-5 (pre-treated with chemo drugs like ARA, doxorubicin, etoposide or vincristine), AML cells showed an increase of mitochondrial content by 14% and resulted in a higher survival of leukemic blasts and leukemia initiating cells [43].

THP-1 cells were known to supress T cells and this suppressive ability of THP-1 cells was reversed by blocking LILRB4 signaling by monoclonal antibody, h128-3. This has been elucidated by a co-culture model of THP-1 cells and T cells. In addition to this, co-culture of peripheral blood mononuclear cells (PBMCs) and THP-1 cells in presence of h128-3, led to the increased secretion of cytokines such as IL2, IL7, CXCL9 and CXCL11, which help in the proliferation and activation of T cells [44]. LILRB4, is an immunoreceptor tyrosine-based inhibition motif containing receptor and a marker of monocytic leukemia. It has been shown by co-culture studies with AML cells and T cells that blocking or deletion of LILRB4 signaling inhibited AML growth. LILRB4 helps in tumour cell invasion of AML with the help of an immunosuppressive microenvironment and therefore, it is a powerful target for AML treatment [45].

A protocol was developed by co-culturing T cells and drug treated GFP-labeled AML suspension cells. The effect of drugs (targeting Fat mass and obesity-associated protein (FTO), which is an oncogene) were studied by flow cytometry using absolute counting beads. This co-culture assay allows us to test the effects of various drug combinations and study the innate mechanisms, which influence the immune response. Moreover, it could also help in understanding the combined effect of immunotherapy and chemotherapy [46]. The same research group reported that AML cells when pre-treated with FTO inhibitors, CS1 or CS2 and co-cultured with activated T cells, resulted in the increased killing of AML cells along with decreased expression of LILRB4 [47]. Acute myeloid leukemia initiating cells (LICs) are known to be responsible for the initiation and relapse of AML. It has been reported that LICs can be maintained around 3 weeks, by modeling the tumour niche, using stromal feeder layers and hypoxic conditions. This model offers a reliable, easy, and reproducible niche-based culture system suitable to study chemoresistance of LICs and to screen new therapeutic drugs specifically against LICs [48].

### Significance of 3D culture and 3D co-culture models

Although 2D co-culture models are being used in research, 3D models are much more appropriate and are close to the *in vivo* tumour microenvironment [38]. Tumour-stromal cell interactions play a major role in tumour development and progression. Stromal cells such as endothelial cells and immunocompetent cells contribute to tumour angiogenesis and invasion [49]. It is important to recapitulate the tumour microenvironment *in vitro* to study the biology of tumour as well as to screen various therapeutic drug molecules. This is why researchers have developed 3D culture of cancer cells and they are an excellent model compared to the conventional monolayer culture of tumour cell lines [50–52].

Molecular mechanisms of leukemogenesis have been elucidated to a certain extent with the help of 2D culture and animal models. However, the cellular and microenvironmental components, which help in leukemia cell proliferation and resistance of leukemic stem cells from conventional chemo-radiation therapies, are still difficult to be studied [53, 54]. Three major challenges to study AML are 1) current 2D culture requires growth factors and stromal cells, for the prolonged survival of tumour cells 2) 2D culture cannot offer the natural haematopoietic microenvironment, which is also responsible for the drug resistance of leukemic stem cells and 3) *in vivo* animal models are not exactly like the human tumour microenvironment and are expensive and time consuming. Hence, development of an *ex vivo* 3D model could mimic the tumour microenvironment to study AML [55–57].

**Figure 1:**
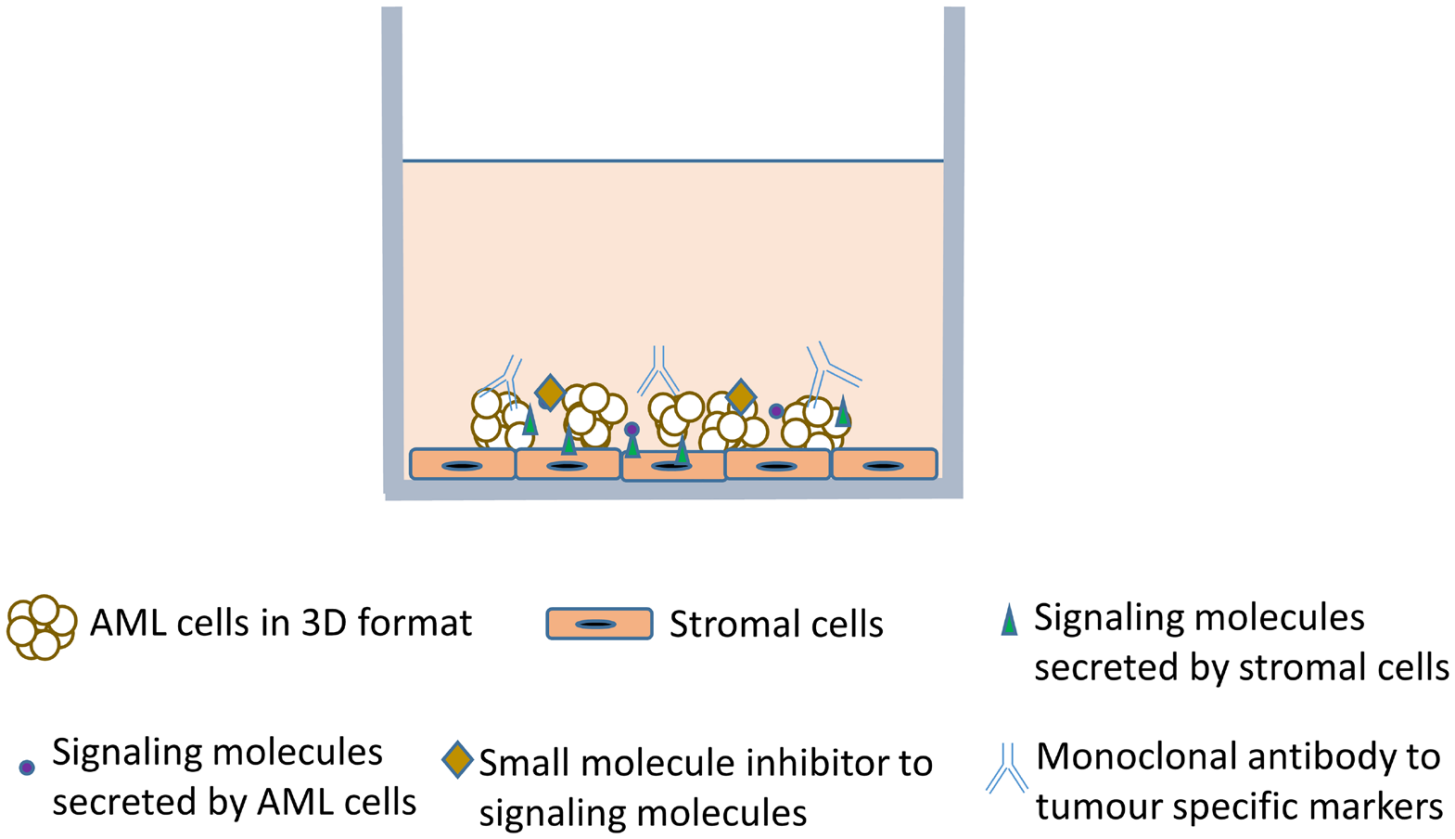
Schematic of a direct 3 Dimensional (3D) co-culture model of acute myeloid leukemia (AML) cells and stromal cells. Small molecule inhibitors or functional monoclonal antibodies targeting tumour specific molecule can be tested for their efficacy using this culture model.

Three dimensional cell culture offers several advantages over conventional 2D culture [58, 59]. The alterations in cell morphology and their adaptive responses, expression of genes and metabolism are similar to that of *in vivo* tumour microenvironment [60]. Cells in 3D culture are more resistant to drugs and therefore, they are physiologically relevant and can better show the drug effects [61, 62]. The cells in the inside core of the tumour are in hypoxic conditions and this holds true with 3D cell culture as well [63]. In 2D culture, proliferation rates, gene and protein expression levels are different from that of the original tumour [64] whereas they are similar to the *in vivo* tumours in 3D culture models [60, 65]. Hence, 3D models would be ideal to study various attributes of tumour such as angiogenesis, metastasis, and invasion, and also in identification of specific biomarkers and screening of drug targets [52, 66]. A schematic of direct 3D co-culture model with AML cells and stromal cells is illustrated in Figure 1 and highlighted in Table 1.

**Figure 2:**
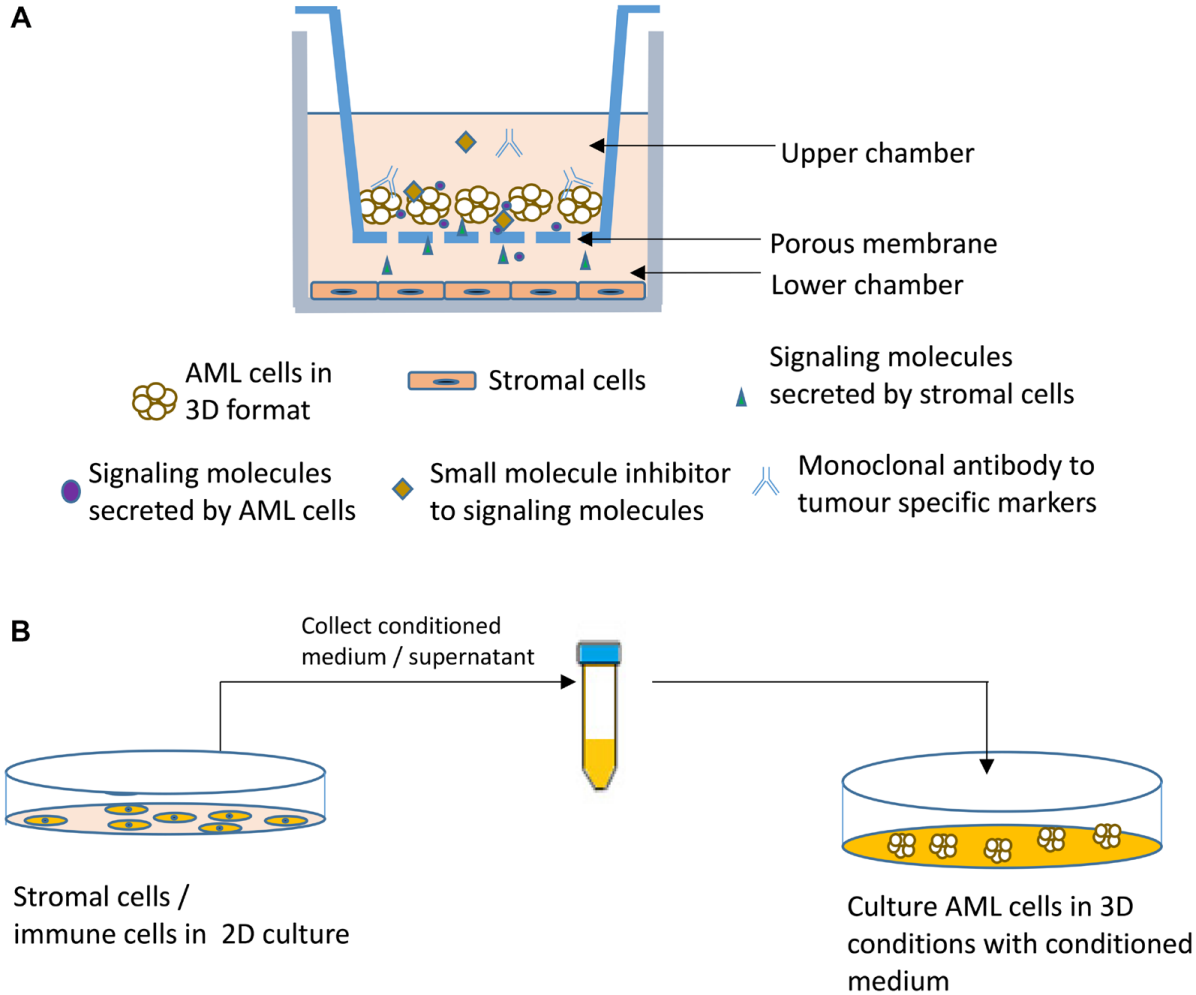
Schematic of two types of indirect 3 Dimensional (3D) co-culture model of acute myeloid leukemia (AML) cells and stromal cells. (**A**) A transmembrane separates the two types of cells and can be collected separately from each compartment and the assays can be done for each type of cells. (**B**) This model can be used for testing different drugs on AML (3D) cells and also the 3D cells can be recovered after the culture. There will be no contamination of stromal/immune cells which is helpful for further down-stream applications.

In order to mimic the histological conditions of a tumour tissue, it is necessary to simultaneously grow tumour and stromal cells with cell-cell interactions and signaling cascades through various growth factors [67]. The presence of an extracellular matrix and interstitial fluid with essential nutrients and growth supplements are required for differentiation and maturation [60, 68, 69]. Three dimensional co-culture models with tumour cells and other stromal cells fulfill these requirements and hence are suitable preclinical tumour models [70, 71]. There are reports on 3D co-culture models to be more efficient to screen drugs. Schematics of indirect 3D co-culture models are illustrated in Figure 2.

### Methods for 3D culture

Broadly, the 3D culture can be divided into two types – 1) Anchorage independent (scaffold free) and 2) Anchorage dependent (scaffold based) [72].

Anchorage independent cultures include methods like hanging drop, ultra-low attachment plate and magnetic levitation. Cells are seeded as small droplets in specialized plates with open, bottom-less wells. Cells in the droplet aggregate and form spherical – like structures. They can be transferred to scaffold or ECM for further prolonged culture [73]. Ultra-low attachment plates are designed such that cells do not attach on their surface and they tend to form aggregates [74]. Magnetic levitation is the technique by which cells are preloaded with magnetic nanoparticles and in the presence of an external magnetic field, cells are floated toward air/liquid interface, thereby cell-cell aggregation occurs to form spherical-like structures [72] .

Anchorage dependent culture mainly includes scaffold-based culture systems. It can be of physical support, ranging from simple mechanical structures to ECM-like matrices. Cells can aggregate, proliferate and migrate on these matrices. Cells would be embedded in the matrix so that they are physically and chemically interacting with the scaffold material. Scaffold can be of synthetic (polyethylene glycol (PEG), polylactic acid (PA) and polyglycolic acid (PGA) or biological origin (alginate and matrigel) [72, 75].

### Challenges in 3D culture

Although 3D culture mimics the *in vivo* tumour conditions, they are quite expensive and time consuming than 2D culture. They are more complex structures and hence downstream applications on these spheroids would need more optimization. Extracting cells from the 3D culture is difficult and sometimes it may change their morphology and original characteristics.

Sometimes, the matrices used in 3D culture would influence the behavior of the cells in 3D culture. It is still challenging to find a matrix or scaffold which exactly matches to that of the *in vivo* tumours. Due to these reasons, it remains challenging to use this model for pre-screening drugs in clinical use.

## FUTURE PERSPECTIVES

Tumour-stromal cell interaction can be studied using 3D co-culture models and these findings can be verified in clinical specimens. Three dimensional culture models in both solid tumours and hematological malignancies, have been a fascinating area of cancer research for more than two decades now and helped in improving our knowledge about the biology of tumours [70].

Some of the current limitations of 2D culture could be overcome by 3D culture and hence novel *in vitro* leukemic models need to be developed. The 3D tumour microenvironment includes different types of cell-cell interactions, cell-extracellular matrix interactions and these factors should be taken into consideration while developing new models [19]. The main objective is to develop a standardized culture model for *in vitro* studies to study the biology of the tumour as well as to test newly developed drug molecules [19]. A recent study reported the use of 3D *ex vivo* culture of CLL cells and showed a substantial increase in proliferative response compared to 2D suspension cultures [76]. This suggests that 3D culture models are more valuable and have more relevance to pathophysiological conditions.

The significance and necessity of co-culture models and 3D co-culture models is very obvious and this area of research needs to be more investigated. Developing *ex vivo* models, which closely mimic the *in vivo* tumour microenvironment should be the major focus. Three dimensional co-culture models with leukemic cells and stromal or immune cells would help to understand the pathogenesis. These models could be relevant in screening various drug targets as well as studying the leukemia initiating cells, which are responsible for the emergence and chemo-resistance of the tumour. Thus, 3D co-culture models could be used as the initial template to screen any drug prior to testing them in an *in vivo* model.
